# What Is the Cause of Toxicity of Silicone Oil?

**DOI:** 10.3390/ma15010269

**Published:** 2021-12-30

**Authors:** Ying Chen, Yan Lam Ip, Liangyu Zhou, Pik Yi Li, Yee Mei Chan, Wai Ching Lam, Kenneth Kai Wang Li, David H. Steel, Yau Kei Chan

**Affiliations:** 1 Department of Ophthalmology, The University of Hong Kong, Hong Kong, China; u3007487@connect.hku.hk (Y.C.); ipv2000@connect.hku.hk (Y.L.I.); u3006804@connect.hku.hk (L.Z.); jodielpy@connect.hku.hk (P.Y.L.); u3566277@connect.hku.hk (Y.M.C.); waichlam@hku.hk (W.C.L.); 2 Department of Ophthalmology, United Christian Hospital, Hong Kong, China; lkw856@ha.org.hk; 3 Sunderland Eye Infirmary, Sunderland SR2 9HP, UK; david.steel@newcastle.ac.uk; 4 Bioscience Institute, Newcastle University, Newcastle upon Tyne NE1 7RU, UK; 5 Department of Eye and Vision Science, University of Liverpool, Liverpool L69 3BX, UK

**Keywords:** emulsification, low-molecular-weight component (LMWC), Müller cell (rMC-1), photoreceptor cell (661W), polydimethylsiloxane, retinal toxicity

## Abstract

Purpose: To investigate the toxicity of the low-molecular-weight components (LMWCs) in ophthalmic silicone oils (SilOils) on retinal cell lines. Methods: The toxicity of six types of LMWCs were studied and compared with conventional SilOil 1000 cSt. In vitro cytotoxic tests of LMWCs, in both liquid and emulsified forms, on three retinal cell lines (Müller cells (rMC-1), photoreceptor cells (661W) and retinal pigment epithelial cells (ARPE-19)) were conducted using a transwell cell culturing system. The morphology and viability of cells were assessed by light microscopy and Cell Counting Kit-8 (CCK-8) assay at different time points (6, 24 and 72 h). The ARPE-19 apoptotic pathway was investigated by Mitochondrial Membrane Potential/Annexin V Apoptosis Kit at different time points (6, 24 and 72 h). Results: Apart from dodecamethylpentasiloxane (L5), all liquid LMWCs showed varying degrees of acute cytotoxicity on retinal cell lines within 72 h. Emulsified LMWCs showed comparable cytotoxicity with liquid LMWCs on retinal cell lines. Cyclic LMWCs, octamethylcyclotetrasiloxane (D4) and decamethylcyclopentasiloxane (D5) had significantly higher cytotoxicity when compared with their linear counterparts decamethyltetrasiloxane (L4) and L5 with similar molecular formula. Using ARPE-19 cells as an example, we showed that LMWCs induce the apoptosis of retinal cells. Conclusions: Most LMWCs, in both liquid and emulsified forms, can induce acute cytotoxicity. In addition, cyclic LMWCs are suspected to have higher cytotoxicity than their linear counterparts. Therefore, LMWCs are suspected to be the main cause of the long-term toxicity of ophthalmic SilOil, due to their toxicity and propensity to cause ophthalmic SilOil to emulsify. The amount of LMWCs should be considered as the paramount parameter when referring to the quality of SilOil.

## 1. Introduction

Since the early years of vitrectomy surgery in the 1980s, SilOil became an important tool in the management of complicated vitreoretinal diseases. However, its use has been reduced due to a series of complications including emulsification, cataract, secondary glaucoma, keratopathy, and inflammation [1]. In addition, the possibility of the direct retinal toxicity of SilOil was proposed based on similar histological findings observed in animal studies [2,3] and enucleated human eyes [4,5]. However, the definitive clinical evidence of SilOil retinopathy is still lacking [6]. Mechanical compression, due to the density difference between SilOil and aqueous humour, was proposed to account for the toxicity of SilOil but was later shown to have a negligible effect [7]. The analytical analysis of ophthalmic SilOils from various manufacturers showed that most SilOils contain chemical impurities including low-molecular-weight components (LMWCs), ionic compounds and residual catalysts [8]. Moreover, several studies have suggested that instead of ophthalmic SilOil itself being toxic to ocular tissues, LMWCs may be the actual culprit [8]. LMWCs, also considered as small molecule SilOils, are the building blocks of long-chain ophthalmic SilOil [9]. They are considered the main impurities in conventional ophthalmic SilOil because they are difficult to remove during the purification procedure. Their cytotoxicity has been proposed to be due to the diffusion of these small molecules into cells [10]. Three types of LMWCs including hexamethylsiloxane (L2), octamethyltrisiloxane (L3) and octamethylcyclotetrasiloxane (D4) have been shown to cause severe inflammation and corneal oedema one week after injection into the anterior chamber of rabbits [8]. L2 has also been reported to cause acute toxicity in ocular cell types in another in vitro study [11]. In addition, due to their significantly lower viscosity, LMWCs have a higher possibility of undergoing emulsification and they also increase the frequency of emulsification to occur in long-chain ophthalmic SilOil [12]. The emulsification of SilOil has been suggested to account for several postoperative complications, including secondary glaucoma and unexplained SilOil-related visual loss [13,14], as emulsified SilOil droplets were found in various ocular tissues [13,14,15,16]. Additionally, the phagocytosis of SilOil-like vesicles by macrophages has been observed in patients with SilOil tamponade [17]. Therefore, the direct toxicity of LMWCs (both in liquid and emulsion form), as well as the chronic inflammation induced by the emulsification of long-chain SilOil due to LMWCs could be the potential mechanism of toxicity of SilOil at an intraocular setting.

A recent in vitro study evaluated the short-term cytotoxicity of LMWCs to human ocular cell lines [18]. However, potential limitations were present in the experimental setup and the types of LMWC used, causing the results of the work to be less translatable [19]. SilOils with varying degrees of purification were tested in this study. As other contaminants were included, the results can only show a certain association of toxicity with the less purified SilOil but not pure LMWCs. In addition, due to its high immiscibility with aqueous liquids and a relative density of less than 1, LMWCs would float on the surface of the culture media; thus, there is no direct contact between LMWCs and ocular cell lines if only simple pre-mixing is performed [19].

To address these limitations [18], in this study, an in vitro transwell cell culture system was used. This system allows the retinal cells to be in direct contact with the LMWCs on the apical side, while receiving nutrients from culture media on their basolateral side [11]. LMWCs vary in their polymer chain length and molecular geometry (linear or cyclic). Furthermore, ocular tissue in the vitreous cavity is exposed to LMWC molecules in both liquid and emulsified droplet forms, as SilOil inevitably emulsifies in situ. Therefore, in this study, the short-term ocular toxicity of various types of linear and cyclic LMWCs, in both liquid and emulsified forms, were investigated.

## 2. Materials and Methods

### 2.1. Materials

#### 2.1.1. SilOil

Four linear LMWCs, namely methyldisiloxane (L2) (#52630, MW = 162.38), octamethyltrisiloxane (L3) (#469319, MW = 236.53), decamethyltetrasiloxane (L4) (#235679, MW = 310.69), dodecamethylpentasiloxane (L5) (#447269, MW = 384.84) and two cyclic LMWCs, octamethylcyclotetrasiloxane (D4) (#235695, MW = 296.62) and decamethylcyclopentasiloxane (D5) (#444278, MW = 370.77) were tested in this study. Silicone oil with a viscosity of 1000 cSt is one of the most commonly used SilOils in ophthalmic surgeries [20]. Therefore, silicone oil 1000 cSt (1000 cSt SilOil) was used as the control SilOil (#378399). These materials were all purchased from Sigma Aldrich (St. Louis, MO, USA).

#### 2.1.2. Retinal Cell Lines

Three eye cell lines, including photoreceptor cells (661W),retinal Müller cells (rMC-1) and retinal pigment epithelial cells (ARPE-19) were used. The 661W cells were immortalised murine cone cells used at passage number 26–30 [21]. rMC-1 is an immortalised rat Müller cell line which was derived from the retinal tissue of adult rats [22]. rMC-1 at passage 30–35 was used. ARPE-19 is an immortalised cell line which was derived from the retinal tissue of human. Ref. [23] ARPE-19 at passage number 10–18 was used. Both 661W cells and rMC-1 cells were maintained in Dulbecco’s modified Eagle’s media (DMEM; Gibco, Life Technologies Corp., Carlsbad, CA, USA) and supplemented with 10% foetal bovine serum (Gibco, Waltham, MA, USA), 100 U/mL penicillin and 100 µg/mL streptomycin (Gibco). ARPE-19 cells were maintained in Dulbecco’s modified Eagle’s media/Nutrient Mixture F-12 (DMEM; Gibco, Life Technologies Corp., Carlsbad, CA, USA) and supplemented with 2% foetal bovine serum (Gibco), 100 U/mL penicillin and 100 µg/mL streptomycin (Gibco).

### 2.2. Preparation and Characterisation of Emulsified LMWC Droplets

Our previously published methodology was used to produce SilOil emulsions [24]. Mixtures of each LMWC and 0.1% bovine serum albumin (BSA) in 1XPBS in a ratio of 1:99 was subjected to high-speed homogenisation (T 10 basic ULTRA-TURRAX^®^, IKA) under 30,000 rpm/min for 30 s to form SilOil emulsions. Since only a small portion of SilOil clinically emulsifies inside the eye, the 1:99 ratio of LMWCs to aqueous phase was used to mimic the in vivo scenario with 1% of the SilOil content being emulsified within the eye. In reality, this is probably more significantly severe than would occur in most clinical situations. Both the droplet number and size distribution profile of each LMWC emulsion were characterised using a Coulter counter Multisizer^®^4 (Beckman Coulter, Brea, CA, USA). A stable emulsion should have both its droplet number and size distribution remain unchanged during the experimental period (i.e., 72 h in the present study).

### 2.3. The In Vitro Biocompatibility Tests

The cells were cultured as monolayers on 12 mm-diameter semipermeable, 0.4 µm-pore-size, 10 µm-thick polyethylene terephthalate (PET) membranes (Costar Transwell; Catalog No.: 3460, Corning, Inc., Corning, NY, USA). The apical side of the transwell membrane was coated with laminin (source) in a concentration of 2 µg/cm^2^ at 37 °C for 2 h before use. After sufficient rinsing with PBS, cells were seeded on the transwell membrane. The cell seeding density of the three cell types for different experimental time points was optimised to prevent the over-confluence of cell growth in the media control group. The results of the optimisation are summarised in Table 1.

All six kinds of LMWCs were included. Cells incubated with culture media and 1000 cSt. SilOil were treated as control groups. After 1 day of incubation, the media on the apical side were removed. Then, 300 µL of LMWCs in either liquid or emulsified form were added to the apical side of the transwell system and cultured with the cells for 6, 24 and 72 h in a humidified chamber with 5% CO_2_ at 37 °C. This achieved direct contact between the test agents and cells that were on the transwell membrane. Glass coverslips were placed on top of the transwell to prevent the evaporation of test samples during the incubation period. The cell counting kit (CCK-8) (ab228554, Abcam, Cambridge, UK) was used to evaluate the cell viability of the experimental groups relative to the control groups. Cell morphology was also compared before and after incubation. All the results collected in this part of study are from three independent experiments with two replicates each.

### 2.4. The Apoptotic Assay for Investigation of Cell Death Pathway

Mitochondrial Membrane Potential/Annexin V Apoptosis Kit (V35116, Thermo Fisher, Waltham, MA, USA) was used to test the two hallmarks of apoptosis–phosphatidylserine (PS) externalisation and changes in mitochondrial membrane potential, on ARPE-19 cells. The annexin V labelled with Alexa Fluor ^®^488 dye can identify apoptotic cells by binding to PS exposed on the outer leaflet of the plasma membrane to show green fluorescence signal. In addition, the MitoTracker^®^ Red dye can indicate the status of the mitochondrial membrane potential by showing red fluorescence signal. After staining ARPE-19 cells with these two dyes in the provided binding buffer, the apoptotic cells showed green fluorescence with decreased red fluorescence, and living cells showed very little green fluorescence and bright red fluorescence under the 530 nm (for green fluorescence) and 630 nm (red fluorescence) bandpass filters. The experiment for the ARPE-19 cell line was conducted one time with duplicated samples as demonstration.

### 2.5. Statistical Tests

One-way analysis of variance (ANOVA) followed by a Bonferroni test was used to evaluate the statistical differences of cell viability among different testing groups. The results of all individual comparisons are summarised in Supplementary Table S1. A *p*-value of <0.05 was considered to be statistically significant.

## 3. Results

### 3.1. Characterisation of Emulsified Droplets

**Figure 1:**
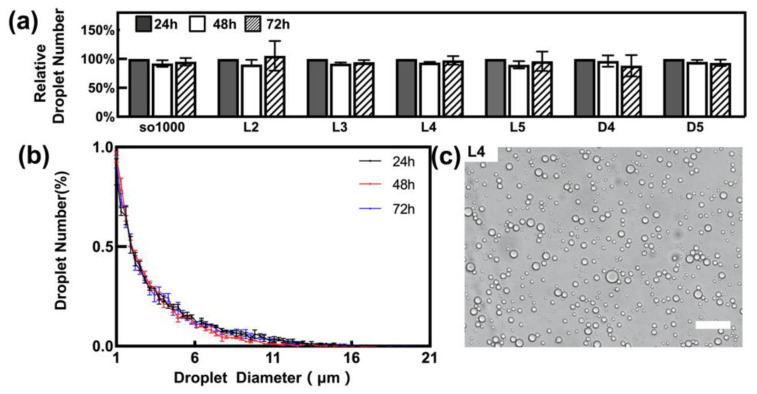
SilOil emulsion generated by high-speed homogenisation: (**a**) the number of emulsified droplets (relative to the number of droplets on Day 1) of various types of LMWC within 72 h after generation; (**b**) the representative emulsified droplets distribution profile of L4 with highly overlapped curves within 72 h after generation; (**c**) the representative morphology of emulsified droplets under fluorescence microscope. Scale bar = 50 μm. The results in (**a**–**c**) are representative of those from 3 independent experiments with 5 replicates each.

The various emulsion samples were stable during the 72 h period after generation by homogenisation. They had a relatively stable total number of droplets (Figure 1a), as well as a similar droplet distribution profile (Figure 1b) during the whole observation period. As shown in Figure 1c, the majority of droplets have a diameter of less than 10 μm, which is observed with light microscopy. In fact, a significantly large proportion of the droplets were less than 5 μm in diameter (Figure 1b), which is comparable to the droplet profile of the droplets in clinical washouts that we have previously reported [25].

### 3.2. Toxicity of Liquid LMWCs on Retinal Cells

**Figure 2:**
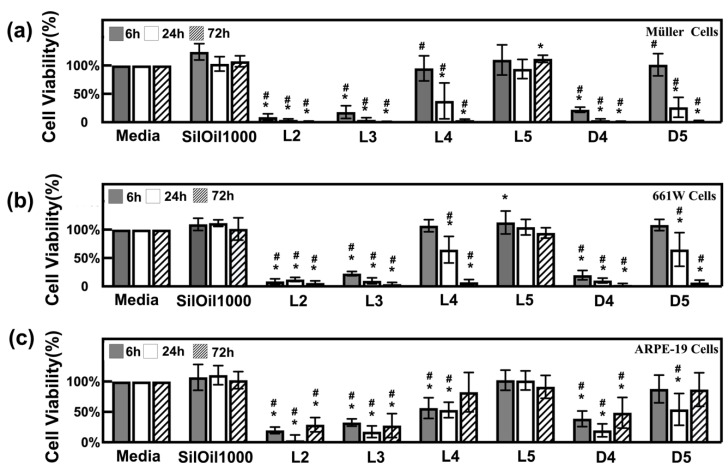
Cytotoxicity of liquid LMWCs. Cell viability of (**a**) Müller cells (rMC-1); (**b**) photoreceptor cells (661W); and (**c**) retinal pigment epithelial cells (ARPE-19) after incubation with various types of LMWCs at different time points. *n* = 6, * *p* < 0.05, vs. media; # *p* < 0.05, vs. SilOil 1000 (one-way ANOVA followed by Bonferroni test). Scale bar = 50 μm. The results in (**a**–**c**) are representative of those from 3 independent experiments with 2 replicates each.

**Figure 3:**
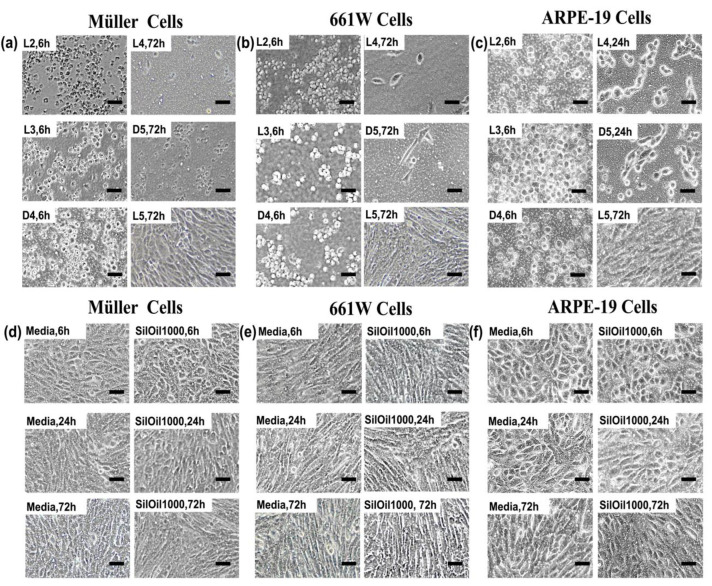
Cell morphology after incubation with liquid LMWCs. Representative images of (**a**) Müller cells (rMC-1); (**b**) photoreceptor cells (661W); and (**c**) retinal pigment epithelial cells (ARPE-19) after incubation with various types of LMWCs at different time points. Morphologies of (**d**) Müller cells (rMC-1), (**e**) photoreceptor cells (661W) and (**f**) retinal pigment epithelial cells (ARPE-19) after incubation with control groups including culture and SilOil 1000 for 6, 24 and 72 h. The results in (**a**–**f**) are representative of those from 3 independent experiments with 2 replicates each.

LMWCs L2, L3 and D4 all displayed acute toxicity to three retinal cell lines rMC-1, 661W and ARPE-19 when compared with the controls (*p* < 0.05). The cell viability rapidly dropped down to 30% within 6 h and further decreased over time (Figure 2a–c). The decrease in cell viability was confirmed by the observation of floating and circular dead cells as shown in Figure 3a–c. LMWCs L4 and D5 exhibited a moderate cytotoxic effect. While these two LMWCs had good compatibility with the cells (rMC-1 and 661W) after 6 h of incubation (*p* > 0.05 when compared with the controls), they exhibited significant toxicity after 24 h of incubation (*p* < 0.05) (Figure 2a,b). For ARPE-19 cells, these two LMWCs exhibited significant toxicity between 6 and 24 h of incubation (*p* < 0.05), while they showed acceptable compatibility after 72 h of incubation. In contrast, L5 showed no significant adverse effects on the retinal cell lines during the observation period as compared with both control groups (Figure 3d–f), in terms of morphology and cell viability (Figure 2a–c and Figure 3a–c) (*p* > 0.05).

### 3.3. Toxicity of Emulsified LMWCs on Retinal Cells

**Figure 4:**
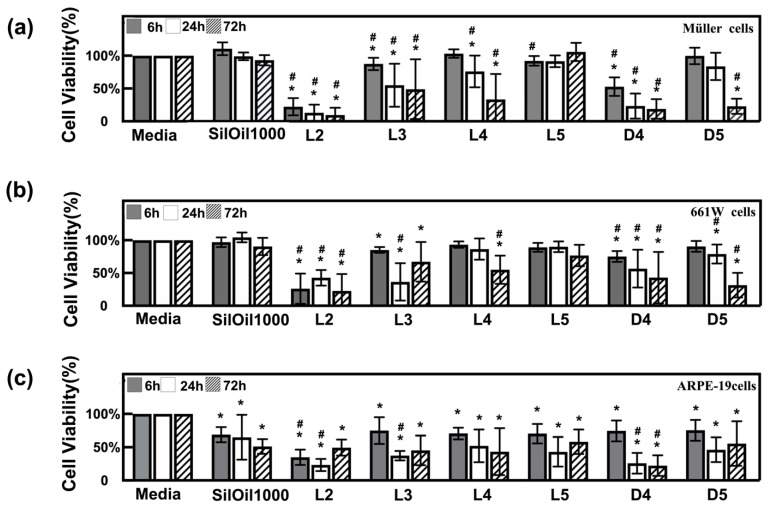
Cytotoxicity of emulsified LMWCs. Cell viability of (**a**) Müller cells (rMC-1); (**b**) photoreceptor cells (661W); and (**c**) retinal pigment epithelial cells (ARPE-19) after incubation with various types of emulsions at different time points. *n* = 6, * *p* < 0.05, vs. media; # *p* < 0.05, vs. SilOil 1000 (one-way ANOVA followed by Bonferroni test). Scale bar = 50 μm. The results in (**a**–**c**) are representative of those from 3 independent experiments with 2 replicates each.

**Figure 5:**
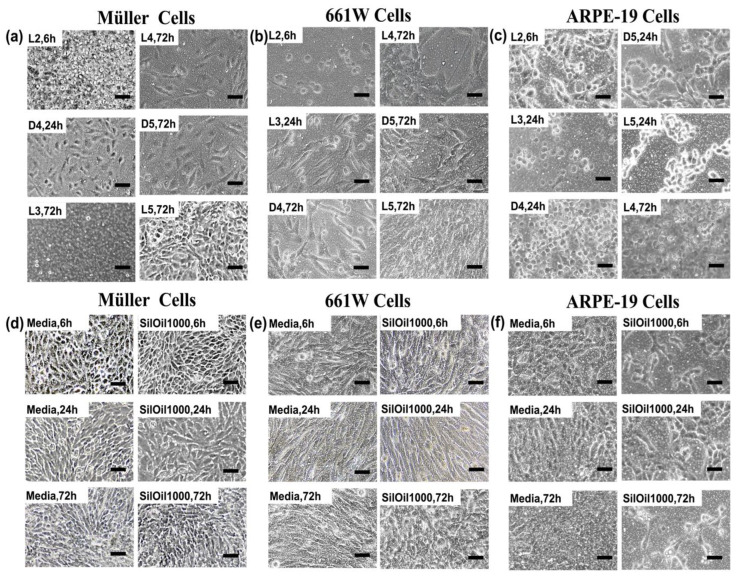
Cell morphology after incubation with emulsified LMWCs. Representative images of (**a**) Müller cells (rMC-1); (**b**) photoreceptor cells (661W); and (**c**) retinal pigment epithelial cells (ARPE-19) after incubation with various types of emulsions at different time points. Morphologies of (**d**) Müller cells (rMC-1); (**e**) photoreceptor cells (661W); and (**f**) retinal pigment epithelial cells (ARPE-19) after incubation with control groups including culture media (media) and emulsion of SilOil 1000 for 6, 24 and 72 h. The results in (**a**–**f**) are representative of those from 3 independent experiments with 2 replicates each.

Emulsions of LMWCs L2, L3 and D4 showed the highest toxicity to all three retinal cell lines. Their cell viability, when exposed to these emulsions, exhibited a significant decline at 6 h, when compared with the controls (*p* < 0.05) (Figure 4a–c). This was further confirmed by the floating and circular dead cells or the attached but shrunken cells, as shown in Figure 5a–c. Emulsions of LMWCs L4 and D5 exhibited a moderate cytotoxic effect on all three retinal cell lines. In particular, they showed a significantly higher toxicity to ARPE-19 than the other two cell lines, even after 6 h of incubation (*p* < 0.05 compared with controls) (Figure 4a–c). In addition, the emulsion of L5 and SilOil 1000 showed a significant difference in toxicity among all three retinal cell lines. They showed no significant adverse effect on two of the retinal cell lines (rMC-1 and 661W) within the observation period as compared with the control group (media) (Figure 5d,e), in terms of both morphology and cell viability (Figure 4a,b and Figure 5a,b) (*p* > 0.05). In contrast, they showed significant toxicity to ARPE-19 during the observation period as compared with the control group (media) (Figure 5f), in terms of both morphology and cell viability (Figure 4c and Figure 5c) (*p* < 0.05). In general, higher cytotoxicity was observed with emulsified LMWCs in ARPE-19 as compared with the other two retinal cell lines (Figure 4 and Figure 5).

### 3.4. Apoptosis of ARPE-19 Cells after Treatment with Liquid LMWCs

**Figure 6:**
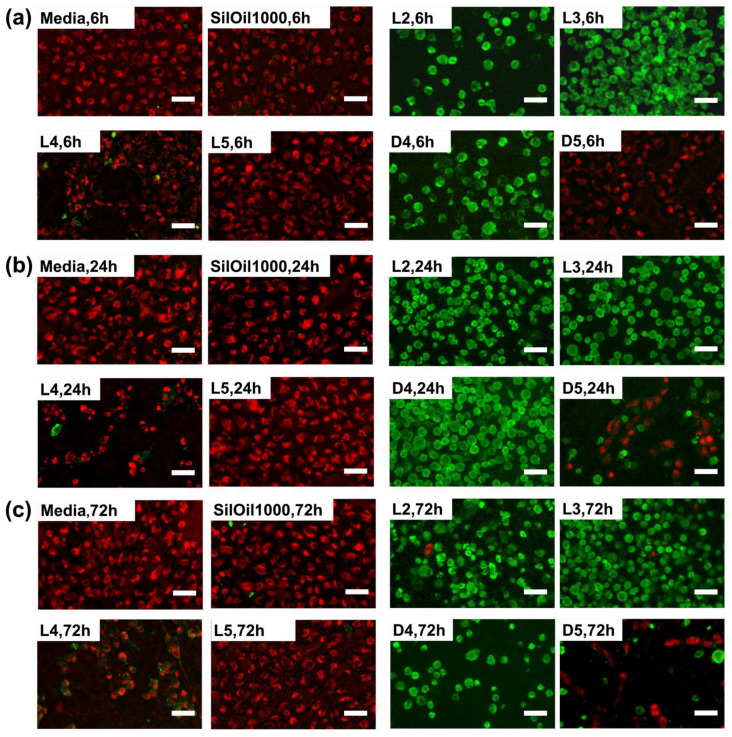
Fluorescent microscopic images of the ARPE-19 cells after incubating with liquid LMWCs and control groups at (**a**) 6 h, (**b**) 24 h and (**c**) 72 h. Cells in red and green represent living and dead cells, respectively. The results in (**a**–**c**) are representative of those from 1 independent experiment with 2 replicates each.

A large amount of floating dead cells with bright green fluorescence was observed after treatment with LMWCs L2, L3 and D4 during the observation periods. In contrast, a large proportion of bright red fluorescence and a small proportion of green fluorescence were observed in the cells in both control groups and L5 group within the observation periods. This indicates that the majority of ARPE-19 cells were normal. A certain proportion of red and green fluorescence was observed, respectively, in the L4 and D5 groups during the observation periods (Figure 6a–c). These results are consistent with the corresponding changes in cell morphology and cell viability (Figure 2c and Figure 3c,f).

### 3.5. Apoptosis of ARPE-19 Cells after Treatment with Emulsified LMWCs

**Figure 7:**
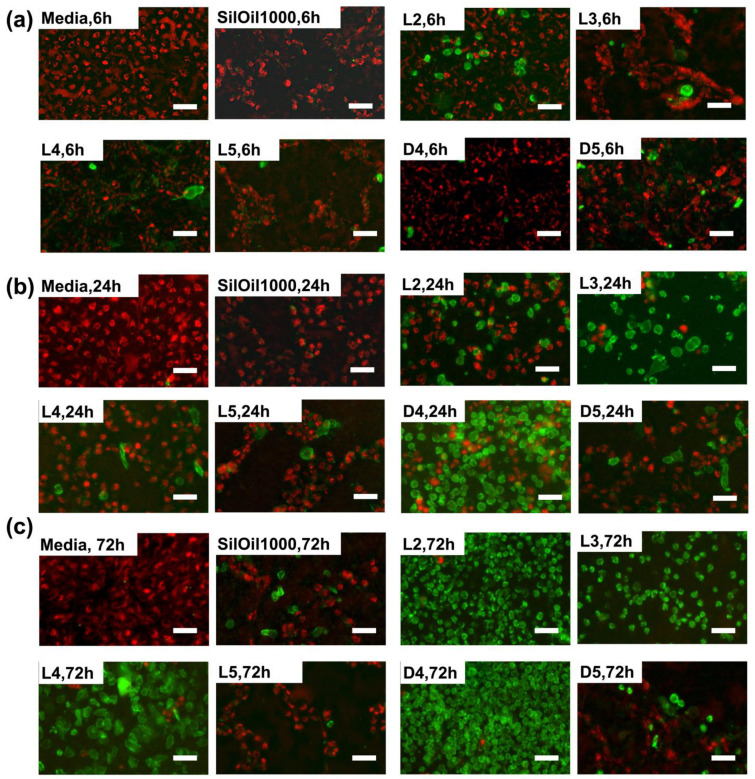
Fluorescent microscopic images of the ARPE-19 cells after incubating with emulsified LMWCs and control groups at (**a**) 6 h, (**b**) 24 h and (**c**) 72 h. Cells in red and green represent living and dead cells, respectively. The results in (**a**–**c**) are representative of those from 1 independent experiment with 2 replicates each.

The floating dead cells in the L2, L3 and D4 emulsion groups with bright green fluorescence increased while the viable cells with bright red fluorescence decreased over time as compared with control group (media). The number of cells with bright red fluorescence decreased in the D4, D5, L4, L5 and SilOil1000 emulsion groups (Figure 7a–c). These results are consistent with the corresponding changes in cell morphology and cell viability (Figure 4c and Figure 5c,f).

## 4. Discussion

SilOil has been used as a long-term endo-ocular tamponade agent since 1962, and is still currently indispensable in the treatment of complicated vitreoretinal diseases [20]. However, the long-term presence of SilOil in the vitreous cavity may cause a variety of complications, especially retinal toxicity. LMWCs, the chemical impurities in SilOil, have been reported to be the potential cause of retinal toxicity [9]. LMWCs are composed of the same repeating dimethyl-siloxane unit as ophthalmic SilOil but with molecular weights of less than 2400 Dalton [9]. There have been concerns over LMWCs in ophthalmic SilOil for some time [9], but the limited studies over the toxicity of LMWCs have not brought concern to more attention. The common standards regarding these impurities have not been established during the production and disclosure of information by manufacturers [26]. Mendichi et al. and Dresp have both shown that different brands of commercially available ophthalmic SilOils contain varying amounts of LMWCs [27,28]. Furthermore, LMWCs have been reported to be the most abundant component by weight in all chemical impurities. Therefore, a comprehensive study on the cytotoxicity of LMWCs to retinal specific cell types is urgently required. In addition, a more detailed understanding of the cytotoxicity caused by LMWCs would aid the understanding of the threat from their presence in ophthalmic oils and facilitate the development of international standards for the manufacturing of ophthalmic SilOil.

The retinal ganglion cell (RGC) layer is the innermost layer of the neural retina, which makes it psychologically closest in position to SilOil. Therefore, RGC is the retinal cell type that is the most likely to be impaired due to the closet contact with SilOil. However, the RGC-5 cell line has been abandoned in vision science research due to reports of their lack of RGC-specific genes and proteins [29] and the contamination of cultures with another photoreceptor cell line 661W [30]. Therefore, the other three available retinal cell lines were used for a cytotoxicity test of SilOil.

In our study, most LMWCs showed acute cytotoxicity with a varying degree on retinal cell lines within 72 h. The cell viability varied with the molecular weight of the LMWC tested, in which LMWCs with smaller molecular weights had more significant adverse effects on viability. The exact mechanism behind the difference in cytotoxicity among various LMWCs is still not well understood. The potential reason could be that LMWCs with a smaller size have a greater ability to pass through the lipid bilayer of cell membrane, leading to an inflammatory response [31]. In addition, cyclic LMWCs (D4 and D5) appear to induce a more acute cytotoxicity than their linear counterparts (L4 and L5) with a similar molecular weight. This finding suggests that the cytotoxicity of LMWCs may not be only physically related (in general, a smaller molecular size makes it easier to diffuse into the cell) [32], but also morphologically related (due to their chemical structure). Furthermore, as the building blocks in the polymerisation of SilOil, cyclic LMWCs have a much higher content than their linear counterparts in commercially available SilOils [28]. Therefore, the presence of cyclic LMWCs in SilOil may be the key risk factor leading to cytotoxicity, which should be taken into consideration in future testing and purification regimes [27].

Although the underlying mechanism of retinal toxicity remains unclear, the emulsification of SilOil was believed to cause inflammatory response and retinal degeneration [13,14]. As lower viscosity indicated a greater propensity to emulsify [33], LMWCs would be the first to emulsify in ophthalmic SilOil. Therefore, in their emulsified form, LMWCs may be more physiologically relevant for the study of cytotoxicity than their liquid form. In our study, the detrimental effect on retinal cells among all emulsion groups of LMWCs were comparable. Furthermore, they exhibited a higher adverse effect on ARPE-19 cells than their liquid counterparts. It should be noted that the amount of LMWCs in the emulsion groups was only a hundredth of the LMWC content of their liquid counterparts. Even with such a small amount of LMWC content in the emulsified samples, the adverse effect on the cells was comparable with their liquid counterparts. We therefore speculate that emulsified LMWCs may be more detrimental than their liquid counterparts in triggering adverse effects in retinal cells and are the main cause of retinal toxicity.

In addition, the emulsion of ophthalmic SilOil—which is significantly more viscous in nature (e.g., SilOil > 1000 cSt)—exhibited a great variation in cytotoxicity to three retinal cell lines. Our results show that the emulsion of SilOil 1000 cSt had no significant cytotoxicity on two retinal cell lines during the experimental period, including the rMC-1 and 661W. This is consistent with the results of another in vitro study, in which emulsions of SilOil 5000 cSt did not induce a severe inflammatory response in neutrophils or stimulate phagocytosis by monocytes when compared with other less viscous ophthalmic liquids [32]. However, it showed an acute adverse effect on ARPE-19 cells during the observation periods. The emulsification of long-chain SilOil is inevitable in the long term and can be greatly aggravated by the presence of LMWCs [12]. In addition, most studies have shown that retinal histological changes occur 6 months after SilOil injection [4,6]. Therefore, the emulsions of long-chain SilOil may also be toxic to retinal tissues during a prolonged period, which requires further long-term experiments for confirmation.

To determine the cell death pathway upon both liquid and emulsified LMWC exposure, specific characteristics associated with apoptosis were investigated. The phosphatidylserine (PS) is a type of amino phospholipid which is exclusively located in the inner leaflet in most viable cells. The migration of PS into cell surface has been considered the universal feature of apoptotic cells [34]. In addition, the loss of mitochondrial membrane potential can be used as additional synergetic biomarkers of apoptosis [35]. Therefore, through combination with Annexin V labelled with a fluorophore, the apoptotic cells with PS exposure in the extracellular environment can be identified. In addition, the presence of mitochondrial membrane potential can be observed through an accumulation of MitoTracker^®^ Red dye in the living cells. In our study, the bright green fluorescence with different proportion was observed in the cells in both liquid and emulsified LMWC groups, indicating that the apoptotic process was involved in the ARPE-19 cell death pathway after LMWC treatment. This is consistent with the results of another in vitro study in which D4 induced the death of Jurkat cells with several apoptotic processes including the cleavage of caspase-3 and DNA fragmentation [36]. However, the underlying mechanism of cell death induced by LMWC exposure still requires further long-term experiments to confirm.

Based on these results, we therefore suggest that the toxicity of SilOil could result from the presence of LMWCs in SilOil and is further aggravated by the emulsification of both LMWCs and long-chain SilOils. In addition, cyclic LMWCs have been quantified as the most abundant impurities in SilOils [28]. Therefore, a more complete removal of LMWCs, particularly the more toxic cyclic form of LMWCs, is urgently needed during manufacture. The amount of LMWCs should be considered as the paramount parameter when referring to the quality of SilOils. Moreover, manufacturers of ophthalmic SilOils should reach an agreement to unify the regulatory standards for both productions of ophthalmic SilOil, as well as information on the constituents of the SilOil that needs to be disclosed in the final product. In particular, manufacturers should specify the concentration of cyclic LMWCs present in their marketed products, making it transparent for vitreoretinal surgeons to choose the highest purity products. Without this knowledge, it will be impossible for surgeons to draw any conclusions regarding the origin of SilOil-related complications in clinical practice [26].

There are several limitations to this study. The cytotoxicity of LMWCs was only studied in the short-term (i.e., up to 3 days). Given the routine longer-term use of SilOil intraocularly, longer-term investigations are required. Furthermore, only three isolated retinal cell lines were evaluated to more comprehensively evaluate LMWC toxicity, further long-term studies, including ex vivo and in vivo animal studies, will be necessary. In addition, only the ARPE-19 cells death pathway was investigated for demonstration to further investigate the underlying mechanism of cell death induced by LMWCs, distinct biochemical signalling associated with the apoptosis of retinal cell lines needs to be comprehensively studied.

## 5. Conclusions

In this study, we showed the short-term cytotoxicity of LMWCs in both liquid and emulsified forms on retinal cell lines in vitro. The molecular weight and molecular geometry may contribute to the difference in cytotoxicity between the various LMWCs tested. The toxicity of SilOil could result from the presence of LMWCs in SilOil and may be further aggravated by the emulsification of LMWCs and long-chain SilOils. In addition, the emulsions of LMWCs could be the main cause of retinal toxicity observed in clinical cases that were related to SilOil emulsification. The apoptotic process may be partially involved in the retinal cell death pathway induced by both liquid and emulsified LMWCs. In agreement with Steel et al., [26], manufacturers should declare the amount of LMWCs present in their marketed products, making it transparent for vitreoretinal surgeons to choose the highest purity products. In addition, manufacturers should set up regulatory frameworks and share good manufacturing practices to minimise LMWCs, particularly cyclic LMWCs, which is the major cause of toxicity of SilOils, as well as manufacturing practices that reduce emulsification.

## Figures and Tables

**Table 1:** The optimised seeding density (number of cells) of three retinal cell types.

	6 h	24 h	72 h
Photoreceptor cells (661W)	1.0 × 10^5^	5.0 × 10^4^	2.5 × 10^4^
Müller Cell (rMC-1)	9.0 × 10^4^	4.5 × 10^4^	2.0 × 10^4^
Retinal pigment epithelial cells (ARPE-19)	1.2 × 10^5^	7.0 × 10^4^	4.0 × 10^4^

## Supplementary Materials

The following supporting information can be downloaded at: https://www.mdpi.com/article/10.3390/ma15010269/s1, Table S1: Statistical comparison on cell viability between different study groups within observation times (up to 72 h).
