# Supplementary material for: What Is the Cause of Toxicity of Silicone Oil?
Source: Materials (Basel). 2021 Dec 30;15(1):269. doi: 10.3390/ma15010269 (PMC8745808; doi:10.3390/ma15010269)
Supplement: Supplementary file 1 [file materials-15-00269-s001.zip › materials-1401708-supplementary.pdf]

Supplementary Table S1. Statistical comparison on cell viability between different study groups within observation times (up to 72 hours)

Cell Types	Forms	Time points	L2	L3	L4	L5	D4	D5	Control Groups
rMC-1	Liquid Form	6hours	<0.0001	<0.0001	>0.9999	>0.9999	<0.0001	>0.9999	Media
		24hours	<0.0001	<0.0001	<0.0001	>0.9999	<0.0001	<0.0001	
		72hours	<0.0001	<0.0001	<0.0001	<0.0001	<0.0001	<0.0001	
		6hours	<0.0001	<0.0001	0.0002	0.35	<0.0001	0.01	SilOil1000
		24hours	<0.0001	<0.0001	<0.0001	>0.9999	<0.0001	<0.0001	
		72hours	<0.0001	<0.0001	<0.0001	0.15	<0.0001	<0.0001	
	Emulsified Form	6hours	<0.0001	0.03	>0.9999	0.69	<0.0001	>0.9999	Media
		24hours	<0.0001	<0.0001	0.02	>0.9999	<0.0001	0.39	
		72hours	<0.0001	<0.0001	<0.0001	>0.9999	<0.0001	<0.0001	
		6hours	<0.0001	<0.0001	0.89	0.0002	<0.0001	0.11	SilOil1000
		24hours	<0.0001	<0.0001	0.03	>0.9999	<0.0001	0.51	
		72hours	<0.0001	0.0001	<0.0001	>0.9999	<0.0001	<0.0001	
661W	Liquid Form	6hours	<0.0001	<0.0001	>0.9999	0.04	<0.0001	0.65	Media
		24hours	<0.0001	<0.0001	<0.0001	>0.9999	<0.0001	<0.0001	
		72hours	<0.0001	<0.0001	<0.0001	>0.9999	<0.0001	<0.0001	
		6hours	<0.0001	<0.0001	>0.9999	>0.9999	<0.0001	>0.9999	SilOil1000
		24hours	<0.0001	<0.0001	<0.0001	>0.9999	<0.0001	<0.0001	
		72hours	<0.0001	<0.0001	<0.0001	0.46	<0.0001	<0.0001	
	Emulsified Form	6hours	<0.0001	0.0059	>0.9999	0.11	<0.0001	0.29	Media
		24hours	<0.0001	<0.0001	0.69	>0.9999	<0.0001	0.04	
		72hours	<0.0001	0.01	0.0001	0.19	<0.0001	<0.0001	

ARPE-19	Liquid Form	6hours	<0.0001	<0.0001	<0.0001	>0.9999	<0.0001	0.45	Media
		24hours	<0.0001	<0.0001	<0.0001	>0.9999	<0.0001	<0.0001	
		72hours	<0.0001	<0.0001	0.41	0.99	<0.0001	0.80	
		6hours	<0.0001	<0.0001	<0.0001	0.99	<0.0001	0.03	SilOil1000
		24hours	<0.0001	<0.0001	<0.0001	0.83	<0.0001	<0.0001	
		72hours	<0.0001	<0.0001	0.24	0.94	<0.0001	0.60	
	Emulsified Form	6hours	<0.0001	0.0002	<0.0001	<0.0001	0.0001	0.0002	Media
		24hours	<0.0001	<0.0001	<0.0001	<0.0001	<0.0001	<0.0001	
		72hours	<0.0001	<0.0001	<0.0001	<0.0001	<0.0001	<0.0001	
		6hours	<0.0001	0.97	>0.9999	>0.9999	0.99	0.96	SilOil1000
		24hours	<0.0001	0.0079	0.72	0.07	<0.0001	0.20	
		72hours	>0.9999	>0.9999	>0.9999	>0.9999	0.02	>0.9999	

*Significant and non-significant values are shown in grey and white boxes respectively.
